# A Low-Temperature-Active Pectate Lyase from a Marine Bacterium for Orange Juice Clarification

**DOI:** 10.3390/microorganisms13030634

**Published:** 2025-03-11

**Authors:** Yujing Bai, Jin Wang, Yongliang Yan, Yuhua Zhan, Zhengfu Zhou, Min Lin

**Affiliations:** 1 School of Life and Health Sciences, Hainan University, Haikou 570228, China; yujingbai2022@163.com; 2 National Key Laboratory of Agricultural Microbiology, Biotechnology Research Institute, Chinese Academy of Agricultural Sciences, Beijing 100081, China; wangjin@caas.cn (J.W.); yanyongliang@caas.cn (Y.Y.); zhanyuhua@caas.cn (Y.Z.); 3 Key Laboratory of Agricultural Microbiome (MARA), Biotechnology Research Institute, Chinese Academy of Agricultural Sciences, Beijing 100081, China

**Keywords:** Pel1Ba, low-temperature-active, juice clarification, marine bacterium

## Abstract

Cold-adapted pectin lyases are particularly useful in the extraction and clarification of freshly squeezed fruit juices at low temperatures, as they effectively reduce juice viscosity and improve light transmittance. With the increasing attention on low-temperature pectinase in industrial applications, the exploration of low-temperature pectinase with novel characteristics has become one of the key focuses of research and development. In this study, a 1026 bp gene, *pel1Ba*, encoding a 42.7 kDa pectin lyase, was cloned from sediment samples collected from the South China Sea and heterologously expressed in *Escherichia coli.* The purified Pel1Ba exhibited an optimal temperature of 40 °C and an optimal pH of 10, with a total enzyme activity of 5100 U/mL. Notably, Pel1Ba is a cold-adapted enzyme that retains 80% of its relative activity across the temperature range of 0–40 °C. When 20 U/mL purified Pel1Ba was added to orange juice, the juice volume increased by 43.00% and its clarity improved by 37.80%. Meanwhile, site-directed mutagenesis analysis revealed that the residual enzyme activities of the mutants A230I, F253I, and L292I were increased by 22.5%, 34.4%, and 25.1%, respectively, compared to the wild type. This study concludes that the cold-active pectate lyase Pel1Ba exhibits potential for applications in the food industry.

## 1. Introduction

Pectin is a natural, high-molecular-weight, complex polysaccharide primarily composed of galacturonic acid residues linked by α-(1,4)-glycosidic bonds with varying degrees of esterification. It is a major component of plant cell walls [1]. Pectinases are a group of enzymes that hydrolyze pectin substrates and act on the complex sugar chains in the smooth and hairy regions of pectin, ultimately facilitating complete hydrolysis. Pectinases are widely found in higher plants and microorganisms and play critical roles in biological processes such as plant cell wall expansion and tissue maturation [2].

Due to their complexity, pectic polysaccharides can only be degraded via the combined action of multiple pectinases. Depolymerization of the polygalacturonic acid backbone is achieved through the action of lyase or hydrolase. Pectate lyase (PL, Endo-/EC 4.2.2.2, Exo-/EC 4.2.2.9) cleaves α-(1,4)-glycosidic bonds in polygalacturonic acid through a β-elimination reaction, leading to the formation of unsaturated double bonds by removing a hydrogen atom at the C5 position of galacturonic acid residues. Pectate lyases are highly efficient at breaking the α-(1,4)-glycosidic bonds in the main chain of pectin and generate low-toxicity pectic oligosaccharides, offering advantages such as few byproducts, mild reaction conditions, and high efficiency.

Currently, various pectate lyases with application potential have been identified in bacteria and fungi. Truong et al. [3] isolated the psychrophilic bacterium *Pseudoalteromonas* from Antarctic marine ice and identified two pectate lyases exhibiting maximum activity at 30 °C and pH 9–10. The deduced amino acid sequence of the two ORFs showed homology to pectate lyases from *Erwinia chrysanthemi* (PelA) and *Aspergillus nidulans* (PelB). Zhou et al. [4] isolated and characterized the pectate lyase BsPel-PB1 from *Bacillus subtilis.* The optimal temperature for BsPel-PB1 activity was determined to be 50 °C. The enzyme exhibited an optimal pH of 9.5 and demonstrated broad pH stability ranging from pH 5 to 11. The *K*_m_ and *V*_max_ values were 0.312 mg/mL and 1248 U/mL, respectively. Margesin et al. [5] cloned a cold-active pectate lyase PL from psychrophilic yeast, which was produced by the Alpine *Mrakia frigida* strain A15 and the Siberian *M. frigida* strain AG25. The enzymes achieved maximum production at cultivation temperatures of 1 °C or 5 °C. Both crude enzymes retained activity at 30 °C and within a pH range of 5 to 9.

Pectinases have broad applications in juice extraction and clarification, where they effectively reduce juice viscosity and enhance transparency. Enhancing the stability of low-temperature pectate lyases allows these enzymes to remain effective under low-temperature conditions, preventing nutrient loss, flavor changes, or other adverse effects caused by high-temperature treatments. Orange juice contains a high level of pectin, which is one of the main components responsible for juice cloudiness. By studying the clarification of pectin in orange juice, the performance of the enzyme can be effectively evaluated. At the same time, orange juice is one of the most common and representative juices in the juice industry, as well as one of the most widely consumed juices worldwide. The demand for clarified orange juice is very common in the juice processing industry. Therefore, this study chose orange juice as the research object, which has significant practical application value.

Regarding the main pectinases used in the extraction and clarification of fruit juices in industry, the pectinase products available on the market are all enzyme complexes, such as hemicellulase and cellulase. Common pectinases are mostly derived from fungi (e.g., *Saccharomyces cerevisiae*, *Aspergillus niger*) or bacteria (e.g., *Bacillus subtilis*), and these enzyme formulations are typically produced by commercial enzyme manufacturers such as Novozymes, DSM, and DuPont. These enzyme preparations help to degrade pectin and reduce juice viscosity and are widely used in juice production. There are no commercial pectin lyases on the market, only enzyme complexes containing pectinases and cellulases. Additionally, according to the EU Council’s regulations, the Fruit Juice Directive (Council Directive 2001/112/EC) prohibits the use of rennet and cellulase. 

In this study, we identified a new pectate lyase, Pel1Ba, from marine sediment samples collected at a depth of 1700 m in the South China Sea. Pel1Ba shares 94.2% homology with a pectate lyase from *Bacillus subtilis*, and it has not been reported before. Pel1Ba exhibits excellent low-temperature stability, maintaining over 80% enzyme activity under conditions of 20–30 °C, making it suitable for juice processing under low-temperature conditions.

## 2. Materials and Methods

### 2.1. Plasmids, Strains, Chemicals, and Media

Soil DNA was extracted from a sediment sample (2018CK-GC02) collected at a depth of 1700 m in the South China Sea (E 117°56.2877′, N 20°59.8047). The pectin lyase gene *Pel1Ba* was obtained from this DNA. Primer synthesis and DNA sequencing were conducted by Sangon Biotech Co., Ltd. (Shanghai, China). The Pel1Ba coding sequence was synthesized by Genewiz (Suzhou, China). The plasmid pET28a was double-digested with two restriction enzymes (*Sal* I-HF and *Sac* I-HF) to obtain linearized pET28a. Using a related kit from Vazyme, the ligation reaction was set up with a molar ratio of target fragment to linearized vector of 2:1. The mixture was incubated at 50 °C for 45 min to complete the ligation. The recombinant plasmid was then transformed into DH-5α competent cells, and positive clones were screened on solid media containing kanamycin. The sequencing-verified plasmid was subsequently transformed into *E. coli* BL21 (DE3) for selection on solid media containing the appropriate antibiotic. The recombinant cells were cultured in Luria Bertani (LB) medium supplemented with kanamycin (50 μg/mL) at 37 °C with shaking. Citrus pectin was purchased from Yuanye Bio-Technology Co., Ltd. (Shanghai, China). Kanamycin, imidazole, and isopropyl β-D-thiogalactopyranoside (IPTG) were obtained from Sigma-Aldrich (St. Louis, MO, USA). All other chemicals used in buffer and medium preparation were of analytical grade.

### 2.2. Sequence Analysis and Homology Modeling

Protein sequence similarity was analyzed using BLAST (https://blast.ncbi.nlm.nih.gov/Blast.cgi) (accessed on 8 February 2024). Multiple sequence alignments were performed using Clustal X with default parameters, and the results were visualized with ESPript. Homology modeling of Pel1Ba was conducted using the AlphaFold server (https://deepmind.google/technologies/alphafold/alphafold-server) (accessed on 26 February 2024). Nucleotide sequence analysis, molecular weight prediction, and isoelectric point (pI) prediction were conducted using Vector NTI 10.0. BLAST searches were performed on the NCBI database. Molecular weight and pI predictions for Pel1Ba were carried out using the ExPASy-ProtParam online platform (https://web.expasy.org/protparam/) (accessed on 23 April 2024). Phylogenetic analysis was conducted using MEGA 7.0.21 software. A molecular phylogenetic tree of PEL sequences was constructed using the neighbor-joining method to examine the relationships between Pel1Ba and other pectin lyases.

### 2.3. Expression and Purification of Pectin Lyase Pel1Ba

*E. coli* BL21(DE3) cells harboring the pET28a-*Pel1Ba* plasmid were streaked on LB agar plates containing 50 μg/mL kanamycin and incubated overnight at 37 °C. Single colonies were inoculated into liquid LB medium with 50 μg/mL kanamycin and cultured overnight at 37 °C. Colonies were validated by PCR and sequencing (Genewiz). Correctly sequenced strains were preserved in glycerol stocks and plated on LB agar containing kanamycin for further experiments.

Protein expression was induced by adding IPTG (final concentration 0.4 mM) to *E. coli* BL21(DE3) cells harboring pET28a-*Pel1Ba* in LB medium supplemented with kanamycin. Cultures were grown at 37 °C and 220 rpm until OD_600_ = 0.6, followed by induction at 20 °C for 20 h. Bacterial cells were harvested by centrifugation at 8000× *g* for 10 min at 4 °C, washed with PBS, and resuspended in buffer containing protease inhibitors (Sigma). Cells were disrupted using a sonicator (Branson, Brookfield, CT, USA), and cell debris was removed by centrifugation at 8000× *g* for 40 min at 4 °C. The recombinant Pel1Ba was expressed as an N-terminal His-tag fusion protein and purified using Ni-NTA affinity chromatography. Proteins were eluted using a linear imidazole gradient (10–100 mM). Desalting and concentration were performed using an Amicon Ultra 30 kDa centrifugal filter. Protein purity and molecular weight were assessed by 15% SDS-PAGE, and protein concentration was determined using a BCA protein assay kit.

### 2.4. Enzyme Activity Assay

Pectin lyase activity was determined using the A_235_ method by monitoring the increase in unsaturated bonds at 235 nm. The reaction mixture contained 190 μL of 50 mM Gly-NaOH buffer (pH 10.0) with 0.2% citrus pectin, 0.1 mM CaCl_2_, and 10 μL of appropriately diluted enzyme solution. Reactions were conducted at 50 °C for 10 min and terminated by the addition of 300 μL of 30 mM H_3_PO_4_. The absorbance of the reaction products was measured at 235 nm using a Ultramicro Spectrophotometer (NanoPhotometer-NP80, Implen GmbH, Munich, Bavaria, Germany). One unit of pectin lyase activity was defined as the amount of enzyme required to produce 1 μmol of unsaturated bonds per minute, with a molar extinction coefficient of 4600 M^−1^ cm^−1^. All activity measurements were performed in triplicate.

### 2.5. Biochemical Characterization of Pel1Ba

The optimal pH of Pel1Ba was determined by measuring the enzyme activity at 50 °C in buffers with various pH values, including 50 mM Citric acid-Na_2_HPO_4_ buffer (pH 1.0–7.0), 50 mM Tris–HCl buffer (pH 7.0–9.0), and 50 mM Gly–NaOH buffer (pH 9.0–12.0), with 0.2% citrus pectin (*w*/*v*) and 0.2 mM CaCl_2_. The optimal temperature was determined by conducting the reaction at 0–90 °C in standard reaction buffer (50 mM Gly-NaOH pH 10.0) with 0.2% citrus pectin and 0.1 mM CaCl_2_ for 10 min.

pH stability was assessed by incubating the enzyme in 50 mM Citric acid-Na_2_HPO_4_ buffer (pH 1.0–7.0), 50 mM Tris–HCl buffer (pH 7.0–9.0), and 50 mM Gly–NaOH buffer (pH 9.0–12.0) at 20 °C for 120 min. Thermal stability was evaluated by incubating enzyme solutions (0.1 mg/mL) in 50 mM Gly–NaOH buffer (pH 10.0) at 20, 30, 40, and 50 °C for up to 120 min. Residual activity was measured at 20 min intervals. The enzyme was incubated in thin-walled PCR tubes (Axygen, Milford, CT, USA) in a temperature-controlled workstation (NaCha) for precise temperature regulation.

### 2.6. Effect of Metal Ions and Organic Solvents on Pel1Ba Activity

The presence of metal ions can influence enzyme activity through various mechanisms. To evaluate the effects of metal ions on Pel1Ba activity, the enzyme was incubated with 1 or 10 mM Mg^2^⁺, Mn^2^⁺, Co^2^⁺, Cu^2^⁺, Fe^3^⁺, Fe^2^⁺, Ni^2^⁺, Zn^2^⁺, K⁺, and Ca^2^⁺, which were obtained from soluble salt chlorides, as well as different chemical reagents, such as sodium dodecyl sulfate (SDS) and ethylenediaminetetraacetic acid disodium salt (EDTA), for 1 h. The residual enzyme activity was then measured, with the activity of the enzyme in the absence of metal ions defined as 100%.

### 2.7. Determination of Kinetic Parameters

The kinetic parameters (*K*_m_ and *V*_max_) of Pel1Ba were determined in 50 mM Gly-NaOH buffer (pH 10.0) using citrus pectin as the substrate at concentrations ranging from 0.1 to 3.2 mg/mL. Reactions were carried out at 50 °C for 10 min, and the A_235_ method was used to measure the reaction rate. The kinetic parameters were calculated via nonlinear regression with GraphPad Prism 5.0 software (http://www.graphpad.com/prism/) (accessed on 23 October 2024). All data are presented as the mean of three independent measurements.

### 2.8. Application of Pel1Ba in Orange Juice Clarification

Orange juice was prepared by homogenizing 132.37 g of oranges with 0.5% (*w*/*v*) ascorbic acid using a household juicer. The juice was then filtered through an eight-layer gauze cloth (League, Beijing, China) to remove residues. The pH and density of the juice were 3.4 and 1.035 g/mL, respectively. Based on prior experiments, Pel1Ba was incubated with 10 mL of orange juice at 20 °C for 60 min. Juice treated with inactivated enzyme (boiled at 100 °C for 10 min) was used as a control. After incubation, the orange juice was filtered through No. 4 filter paper (Whatman, Little Chalfont, UK), and the volume of filtrate obtained after 5 min was measured. The transmittance of the juice supernatant at 660 nm was recorded. All experiments were conducted in triplicate.

### 2.9. Site-Directed Mutagenesis

Single-site mutations in Pel1Ba were predicted using the Preoptem model [6] (https://github.com/BRITian/Preoptem) (accessed on 19 October 2024), a tool that integrates experimental data, statistical models, and machine learning to predict changes in protein stability following mutations, expressed as ΔΔG (kcal/mol). ΔΔG (kcal/mol) > 0 indicates reduced protein stability, while ΔΔG (kcal/mol) < 0 suggests increased stability. Seven mutation sites with ΔΔG (kcal/mol) < −0.7 were selected for mutagenesis, and primers were designed accordingly (Table S2). Briefly, site-directed mutagenesis was performed using mutagenic primers to amplify the circular pET28a-*Pel1Ba* plasmid by PCR. The PCR products were purified using an agarose gel purification kit and digested with *Dpn*I. The *Dpn*I-treated products were subsequently transformed into *E. coli* DH5α. Sequencing was performed on plasmids isolated from the transformants using the T7 promoter primer. Finally, the mutant recombinant plasmids were transformed into *E. coli* BL21 (DE3) for expression, and enzyme activity assays were conducted using the supernatants. The same strategy was applied to construct and analyze other mutants.

## 3. Results

### 3.1. Cloning and Sequence Analysis of the Pel1Ba Gene

A 1206 bp fragment encoding a pectate lyase gene, Pel1Ba, was identified and cloned from deep-sea sediment metagenomic sequences. The nucleotide sequence of Pel1Ba shared 94.2% identity with a pectate lyase gene from *Bacillus subtilis* that encodes a polypeptide of 402 amino acids. Using the ExPASy-ProtParam tool (https://web.expasy.org/protparam/) (accessed on 23 April 2024), the molecular weight and isoelectric point (pI) of the mature Pel1Ba protein were predicted to be 42.7 kDa and 6.83, respectively. Sequence alignment revealed 88.84% identity with *Bacillus subtilis subsp. subtilis str. 168* [7] and 68.7% identity with *Paenibacillus kribbensis* Pel [8], both of which are members of polysaccharide lyase family 1 (PL1) in the CAZy database (http://www.cazy.org/Polysaccharide-Lyases.html) (accessed on 22 November 2023).

**Figure 1:**
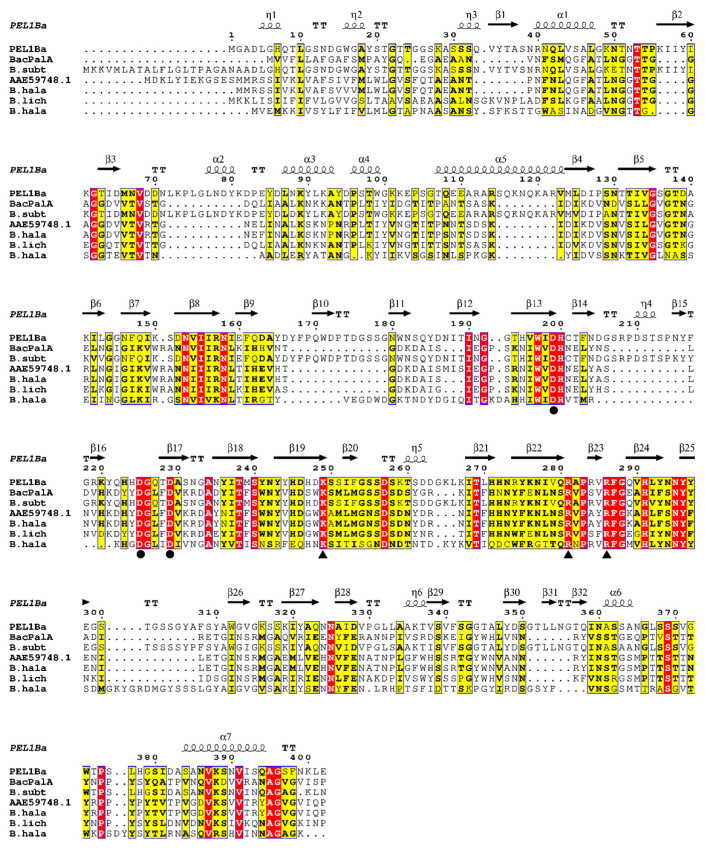
Multiple amino acid sequence alignment. The pectate lyases used were Pel1Ba, BacPelA from *Shouchella clausii* (GenBank no. ALK03050.1), *B. subt* from *Bacillaceae* (GenBank no. CAB12585.1), AAE59748.1 from *Halalkalibacterium halodurans* (GenBank no. AAE59748.1), *B. hala* 1 from *Bacillus sp.* P-4-N (GenBank no. BAB07538.1), *B. lich* from *Bacillus* (GenBank no. CAD56882.1), and *B. hala* 2 from *Halalkalibacterium halodurans* (GenBank no. BAB04417.1),. Strictly conserved residues are shaded red and conservatively substituted residues are shaded yellow. Triangles and circles indicate conserved catalytic sites and calcium-binding sites, respectively. The figure was produced using ESPript 3.0 (http://espript.ibcp.fr/ESPript/ESPript/index.php) (accessed on 6 November 2023).

**Figure 2:**
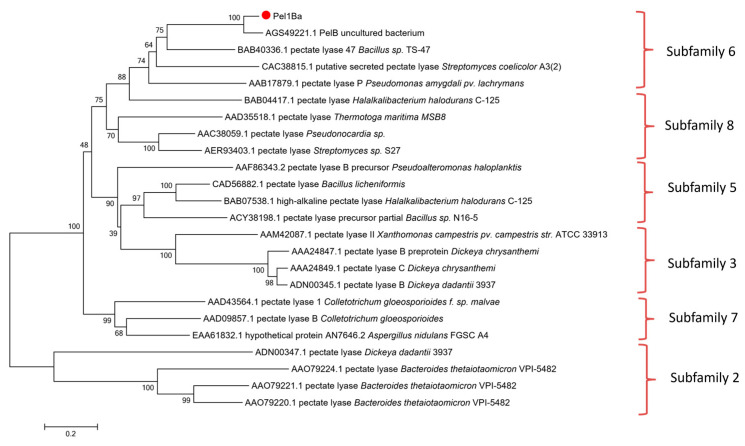
Phylogenetic analysis of the PL1 family.

Three aspartic acid residues (D199, D225, and D229) in Pel1Ba were identified as key calcium-binding residues, analogous to those found in EcPelA (D176, D216, D220) [9] and BsPel (D205, D244, D248) [10]. Essential catalytic residues (K249, R281, R286) in Pel1Ba were also identified on the basis of sequence alignment with previously characterized residues in EcPelE (R240, R245, K212) [11], BsPel (R198, R203, K168) [11], and BsPel (R300, R305, K268) (Figure 1). According to the CAZy database, pectinases are distributed across families PL1, PL2, PL3, PL9, and PL10. To explore the evolutionary trajectory of Pel1Ba and determine its subfamily, we constructed a phylogenetic tree for the PL1 family. The tree revealed that Pel1Ba clustered with representative enzymes from subfamily 6 (Figure 2), indicating that Pel1Ba was a member of the PL1 family, subfamily 6.

### 3.2. Heterologous Expression and Purification of Pel1Ba

**Figure 3:**
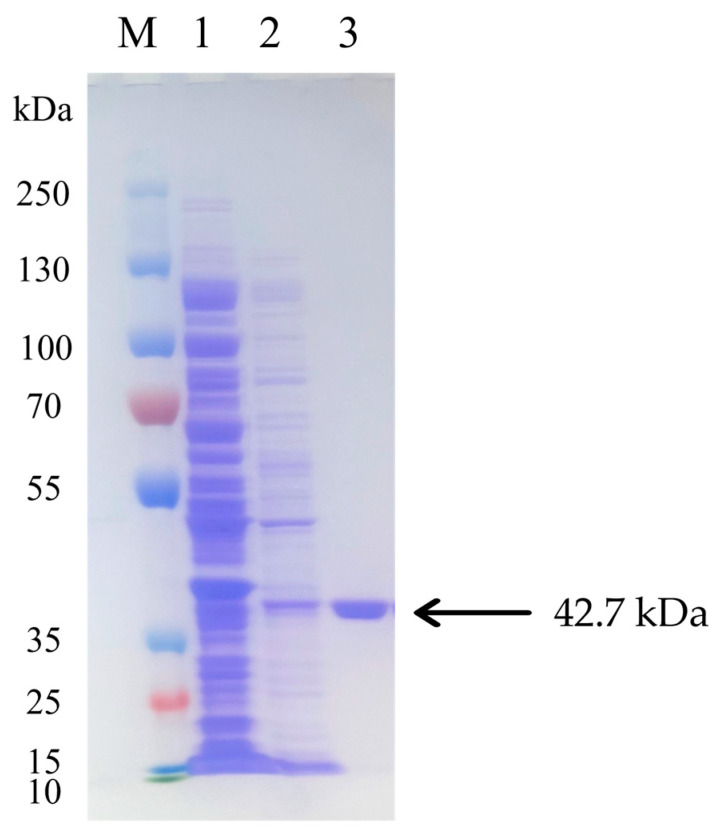
SDS-PAGE analysis of purified Pel1Ba. Lane M, molecular weight marker; Lane 1, purified enzyme Pel1Ba eluted with 40 mM imidazole; Lane 2, purified enzyme Pel1Ba eluted with 80 mM imidazole; Lane 3, purified enzyme Pel1Ba eluted with 100 mM imidazole.

To achieve efficient expression of the mature Pel1Ba enzyme, a prokaryotic expression system, *E. coli* BL21 (DE3)/pET28a-*Pel1Ba*, was constructed. The recombinant strain was cultivated in LB medium using a shake flask system. After 19 h of induction, the cells were disrupted using an ultrasonic cell disruptor. The cell lysate was purified using an NTA column and a 30 kDa centrifugal ultrafiltration device, resulting in a total enzyme activity of approximately 5100 U/mL for Pel1Ba. SDS-PAGE analysis indicated that the purified Pel1Ba from the fermentation supernatant appeared as a single band with a molecular weight of approximately 42.70 kDa (Figure 3), consistent with the theoretical value. The total protein concentration was determined to be 100.96 mg/mL using the BCA method. Under the conditions of pH 10 and 50 °C, the specific activity of Pel1Ba toward 0.2% (*w*/*v*) citrus pectin was 51.30 U/mg, which exceeded the activities reported for two other polygalacturonate lyases using the A_235_ method: a total activity of 52.5 U/mL [12] for *Bacillus subtilis* WSHB04-02 [13] and 360.1 U/mL for *Aspergillus niger* PelA [14].

### 3.3. Effect of Temperature and pH on Enzyme Activity and Stability

**Figure 4:**
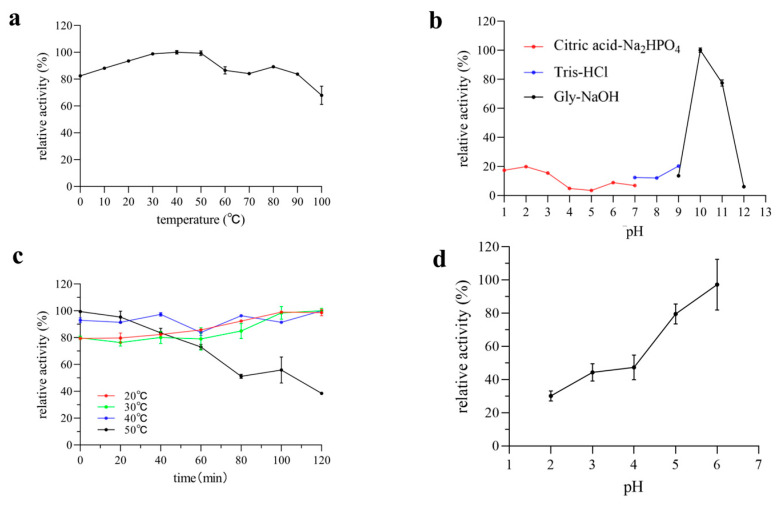
Characterization of purified Pel1Ba. (**a**) Effect of temperature on Pel1Ba activity. (**b**) Effect of pH on Pel1Ba activity. (**c**) Thermostability of recombinant Pel1Ba. (**d**) pH stability of Pel1Ba activity.

pH and temperature are two critical physical factors that influence enzyme activity. The effects of pH and temperature on Pel1Ba activity were investigated using citrus pectin as a substrate. The purified Pel1Ba exhibited an optimal temperature of 40 °C at pH 10, with a relative activity exceeding 30% within the temperature range of 10–50 °C (Figure 4). Remarkably, Pel1Ba retained 80% of its relative activity even at 0 °C (Figure 4). As shown in Figure 4, Pel1Ba maintained most of its activity after incubation at 20 °C, 30 °C, and 40 °C for 120 min. The 20–40 °C range is generally considered mild conditions, and under mild conditions, enzyme activity increases with temperature mainly due to the accelerated molecular motion, which increases the frequency of collisions between the substrate and the enzyme. The flexibility of the enzyme’s conformation is enhanced, and the activation energy is reduced. At moderately elevated temperatures, the enzyme–substrate complex may become more stable. However, at 50 °C, enzyme activity gradually declined, with the residual activity decreasing to 38.77% after 120 min of incubation. The optimal temperature of Pel1Ba is similar to that of PNL from *Curvularia inaequalis* NRRL 13884 (45 °C) [15] but lower than that of PNL from *Penicillium citrinum* (50 °C) [16]. Given that typical juice processing is performed within the temperature range of 30–50 °C, Pel1Ba is well suited for clarifying most types of fruit juices.

The optimal pH for Pel1Ba activity was determined to be pH 10 (Figure 4). Additionally, Pel1Ba retained 20% of its maximum activity at pH 1.0–3.0. After incubation at pH 2.0–4.0 for 120 min at 20 °C, more than 20% of the initial activity was retained, highlighting its suitability for processing acidic fruit juices, such as orange juice (pH 3.4), without requiring pH adjustment.

### 3.4. Effects of Metal Ions and Organic Solvents on Enzyme Activity

Metal ions influence enzyme activity by binding to amino acid residues, with Ca^2^⁺ serving as an activator for many enzymes. This study analyzed the effects of different metal ions and chemical reagents on Pel1Ba at concentrations of 1 mM and 10 mM (Table 1). Among the tested ions and reagents, Mn^2^⁺, K⁺, and Ca^2^⁺ enhanced Pel1Ba activity by more than 20%, suggesting that these ions may serve as cofactors for Pel1Ba catalysis. Conversely, Mg^2^⁺ was the strongest inhibitor, reducing activity by more than 70% at both 1 mM and 10 mM. At 10 mM, Fe^3^⁺ nearly eliminated Pel1Ba activity, resulting in a value of only 2.8%. Additionally, Co^2^⁺ and Cu^2^⁺ inhibited activity by more than 50%.

### 3.5. Kinetic Parameters

The *K*_m_ and *V*_max_ values of Pel1Ba for citrus peel pectin were determined to be 8.978 mol/mL and 0.02997 μmol/min/mg, respectively, with a Kcat of 12.70 s^−1^. The Kcat/Km value was 1.41 × 10^2^ mL·mg^−1^·s^−1^, the decay constant of Pel1Ba was k ≈ 0.0085 min^−1^, and the half-life was t_1_/_2_ ≈ 81.53 min (Figure S1).

### 3.6. Application of Pel1Ba in Orange Juice Processing

**Figure 5:**
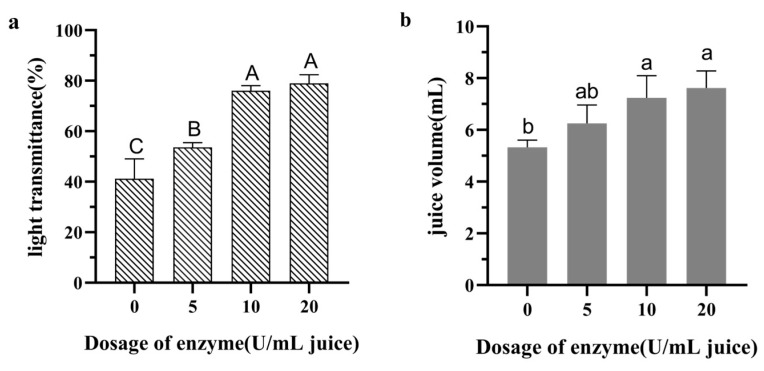
Determination of the optimal amount of purified Pel1Ba for juice clarification.: (**a**) Transmittance of orange juice treated with different dosages of purified Pel1Ba; (**b**) Juice volume after treatment with varying dosages of purified Pel1Ba.

**Figure 6:**
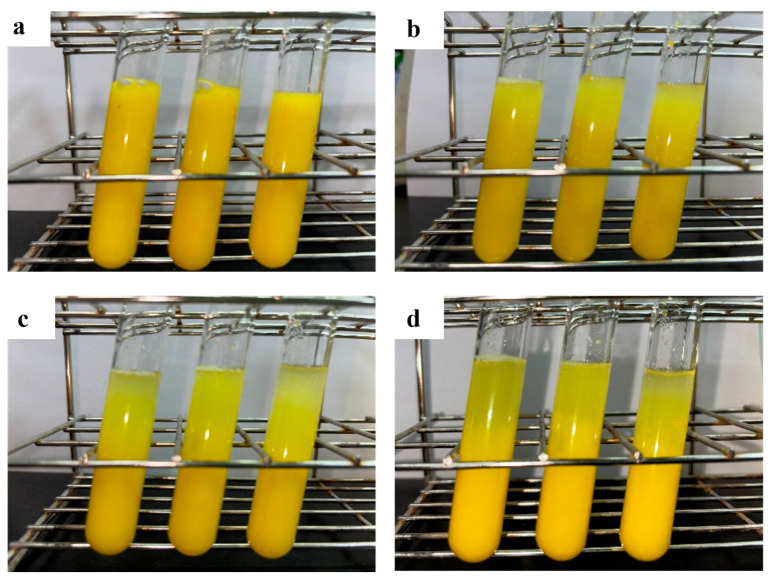
Application of pectate lyase Pel1Ba for fruit juice clarification: (**a**) Effects of 0 U/mL on the light transparency of orange juice; (**b**) Effects of 5 U/mL on the light transparency of orange juice; (**c**) Effects of 10 U/mL on the light transparency of orange juice; (**d**) Effects of 20 U/mL on the light transparency of orange juice.

The pH and density of the juice were 3.4 and 1.035 g/mL, respectively. Pel1Ba demonstrated significant potential in improving the clarity of orange juice. The clarification effect of purified Pel1Ba on orange juice is shown in Figure 5 and Figure 6. Compared with the control, the addition of 5 U/mL of Pel1Ba increased transmittance by 12.42%. Similarly, the transmittance increased by 34.86% and 37.80% with the addition of 10 U/mL and 20 U/mL, respectively. The juice volume also increased, with the volume of 5 U/mL enzyme-treated juice increasing from 5.33 mL (control) to 6.25 mL (a 17.34% increase). Treatment with 10 U/mL and 20 U/mL resulted in increases to 7.24 mL (35.74%) and 7.62 mL (43.00%), respectively. These results indicate that Pel1Ba effectively digested orange pectin and exhibited excellent performance in low-temperature juice clarification.

### 3.7. Structural Modeling and Identification of Catalytic Sites in Pel1Ba

**Figure 7:**
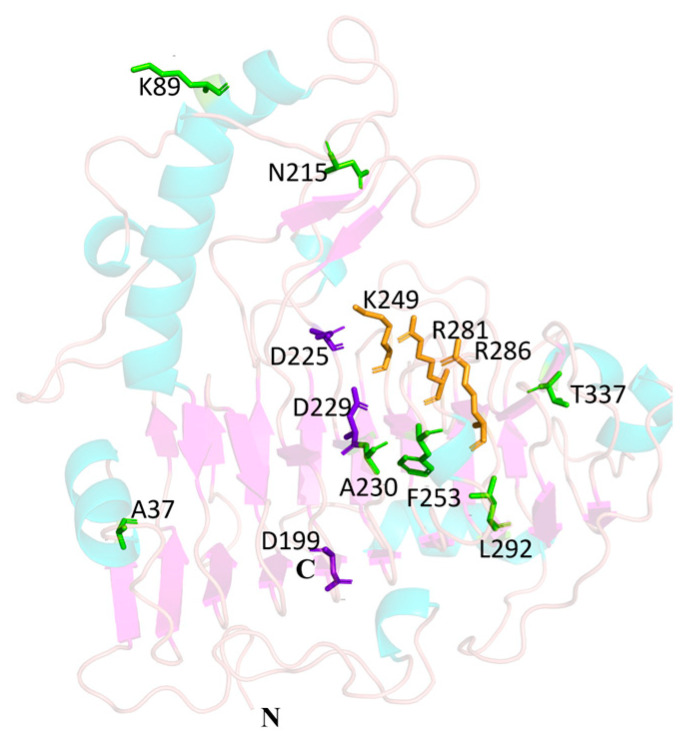
Overall structure of Pel1Ba. Calcium-binding residues are shown in purple, catalytic residues in orange, and the mutated site in green.

The protein structure of Pel1Ba was modeled using AlphaFold 3 software. Its tertiary structure primarily comprised α-helices, irregular loops, and right-handed β-helical folds. The template with the highest homology (pectate lyase BsPel, PDB: 1bn8.1) shared 94.18% amino acid sequence identity with Pel1Ba. The tertiary structure was visualized using PyMOL (Figure 7). Three highly conserved catalytic residues in the PL1 superfamily, K, R, and R, were identified in Pel1Ba as K249, R281, and R286. To verify whether Arg281 was the active site of Pel1Ba, we performed site-directed mutagenesis to substitute Arg281 with Ala. The mutated enzyme, expressed in *E. coli* BL21 (DE3), exhibited no enzymatic activity, indicating that Arg281 was essential for Pel1Ba activity.

### 3.8. Enzymatic Properties of Pel1Ba Mutants

**Figure 8:**
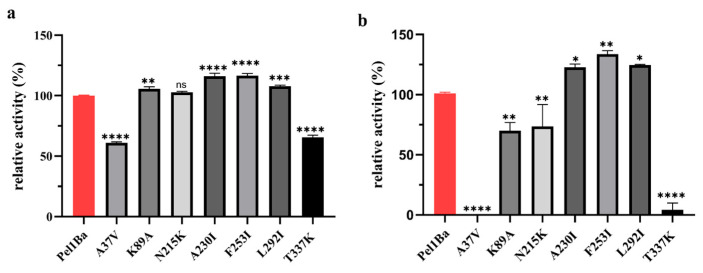
Characterization of Pel1Ba mutants: (**a**) Residual activity of wild-type and mutant Pel1Ba enzymes; (**b**) Temperature stability of mutants at 20 °C. Significance analysis: *p* > 0.05, ns; *p* < 0.05, *; *p* < 0.01, **; *p* < 0.001, ***; *p* < 0.0001, ****.

**Figure 9:**
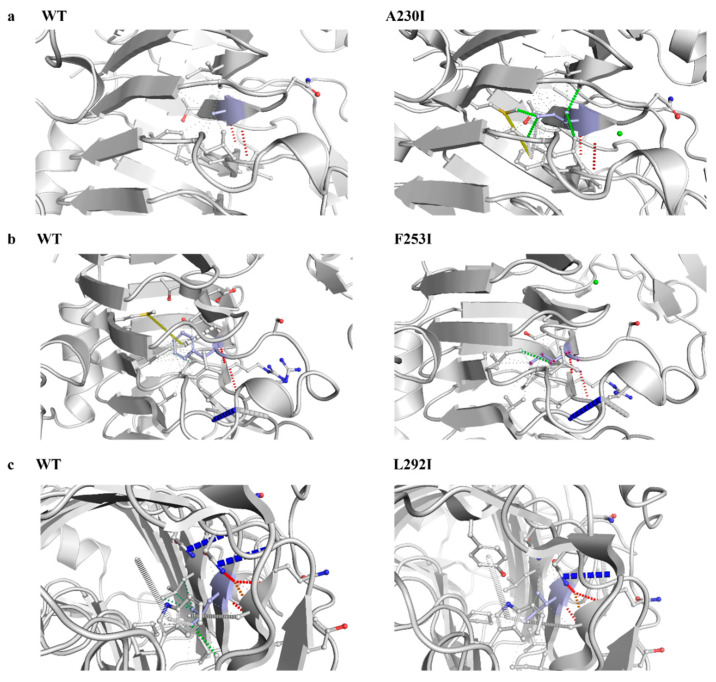
Structural diagrams of amino acid changes at mutation sites. (**a**) Mutation of alanine to isoleucine at site 230 of the pectate lyase Pel1Ba; (**b**) Mutation of phenylalanine to isoleucine at site 253 of the pectate lyase Pel1Ba; (**c**) Mutation of leucine to isoleucine at site 292 of the pectate lyase Pel1Ba.

The Preoptem temperature model was used to predict low-temperature stability. ΔΔG (kcal/mol) > 0: the mutation reduces protein stability (denaturation becomes easier). ΔΔG (kcal/mol) < 0: the mutation increases protein stability. Based on the prediction results, seven mutation sites with ΔΔG (kcal/mol) < −0.7 were selected for mutagenesis. (Tables S1 and S2). Activity assays revealed that the mutants K89A, A230I, F253I, and L292I exhibited significant activity increases of 5.74%, 16.06%, 16.42%, and 7.8%, respectively (Figure 8a). Analysis of the residual activity after 2 h at 20 °C showed that the mutants A230I, F253I, and L292I retained 22.5%, 34.4%, and 25.1% more activity, respectively, than the wild-type enzyme. By contrast, the mutants K89A and N215K showed significantly reduced residual activity, while T337K retained only 4.05% activity, and A37V was entirely inactive (Figure 8b). Structural analysis showed that compared with the wild type, the mutant A230I increased hydrophobic interactions by 23 and reduced carbonyl interactions by 2. The mutant F253I reduced weak hydrogen bonds by 1, hydrophobic interactions by 15, and weak polar contacts by 1. The mutant L292I added a weak hydrogen bond and reduced hydrophobic interactions by 2 and weak polar contacts by 1 (obtained through modeling and computational analysis using the tool DynaMut (https://biosig.lab.uq.edu.au/dynamut/) (accessed on 1 October 2024)) (Figure 9).

### 3.9. Kinetic Parameters of the Mutants

In the enzyme kinetics analysis, wild-type pectinase Pel1Ba was used as a control, and comparison experiments were conducted with the mutants A230I, F253I, and L292I. The resulting enzyme kinetic parameters are shown in Table 2. Compared with the wild-type Pel1Ba, the Km values of the mutants were all reduced, indicating a significant improvement in the enzyme’s affinity for the substrate. The Km values of the mutants A230I, F253I, and L292I decreased by 4.91%, 57.82%, and 10.73%, respectively, compared to the wild type. This phenomenon suggested that the alterations at the mutation sites enhanced the enzyme’s binding capability to the substrate, likely due to the optimization of the enzyme’s substrate-binding pocket or changes in the chemical environment caused by these mutations, thereby increasing the enzyme’s affinity for the substrate. Further analysis of the kcat/Km values revealed that the kcat/Km values of the mutants A230I, F253I, and L292I increased by 15.60%, 41.13%, and 31.91%, respectively, compared to the wild-type Pel1Ba. This change indicated a significant improvement in the efficiency of substrate conversion per unit time for the mutants, with the kcat/Km value of the mutant F253I showing the most substantial increase, highlighting its particularly notable enhancement in catalytic efficiency.

## 4. Discussion

This study revealed that the pectate lyase Pel1Ba possesses broad temperature adaptability, retaining over 80% of its maximum activity at temperatures ranging from 0 °C to 30 °C. Compared with the results for BacPelA reported by Cheng et al. [17], Pel1Ba exhibits broader temperature stability, maintaining over 80% residual enzyme activity at 0 °C, whereas BacPelA retains only 20% residual activity at 50 °C. At 20 °C, the specific activity of Pel1Ba is 43.78% higher than that of BacPelA.

The mutants A37V and T337K are located on β-strands, while the mutants K89A, A230I, F253I, and L292I are located on α-helices. The mutant N215K is located in the loop region. And the F253I mutant exhibited the greatest improvement in stability at 20 °C, with the residual enzyme activity increasing by 34.4% compared with that of the wild type. The hypothesized molecular mechanisms underlying this enhanced stability are as follows: (1) Structural differences between phenylalanine and isoleucine: Phenylalanine is an aromatic amino acid with a bulky and rigid benzyl side chain that occupies substantial space within the protein structure. By contrast, isoleucine is a smaller, nonpolar, and hydrophobic amino acid with an isopropyl side chain, which is more compact and facilitates stronger hydrophobic interactions. The substitution of phenylalanine with isoleucine alters the local structure and hydrophobicity of the protein, potentially enhancing its stability under low-temperature conditions [18]. (2) Enhanced hydrophobicity and low-temperature stability: Hydrophobicity was correlated with the increased low-temperature stability of pectin lyase Pel1Ba [19]. Isoleucine, with its stronger hydrophobic side chain, may improve stability by promoting tighter packing within the protein structure. While phenylalanine also has hydrophobic properties, its bulky benzyl ring can introduce structural flexibility that increases molecular motion. At low temperatures, stronger hydrophobic interactions help to maintain protein stability by reducing conformational freedom and increasing compactness, making isoleucine more favorable than phenylalanine for stabilizing the enzyme structure. (3) Improved conformational stability: Substituting phenylalanine with isoleucine in F253I eliminates one weak hydrogen bond, 15 hydrophobic interactions, and one weak polar contact. These changes improve local interactions between amino acids, reducing structural looseness at low temperatures and enabling the protein to maintain a more stable conformation [20]. (4) Increased folding stability: Isoleucine enhances protein folding stability, thereby decreasing the risk of unfolding under low-temperature conditions. This enhanced rigidity and hydrophobicity support the retention of the folded state of the protein at lower temperatures [21,22]. (5) Improved cold adaptation: The introduction of isoleucine enhances the cold-adaptive properties of Pel1Ba. Cold-adapted enzymes typically exhibit higher hydrophobicity and reduced structural rigidity to maintain activity and stability at low temperatures [23]. Replacing phenylalanine with isoleucine may render the overall structure of Pel1Ba better suited for low-temperature environments, thereby increasing its stability and functionality under cold conditions [24].

In industries such as food processing, feed production, and paper manufacturing—particularly juice processing—the application of exogenous pectinases brings substantial benefits [25]. For instance, the issue of high viscosity in juice can be addressed through the addition of pectinases [26]. Juice extraction can be performed using various mechanical methods; however, the presence of pectin and other polysaccharides in fruit may cause clogging during filtration. Enzymatic treatment degrades pectin, reducing viscosity and promoting the formation of aggregates that facilitate separation through centrifugation or filtration. As a result, the juice exhibits higher clarity, a more concentrated flavor, and enhanced color [27]. Enzymes also contribute to softening plant tissues by hydrolyzing the fruit cell wall, leading to the release of cellular contents, which can then be recovered in higher yields, thereby increasing profitability [28]. Studies have shown that compared with other extraction methods, enzymatic pretreatment prior to mechanical extraction significantly improves juice recovery [29]. Furthermore, enzymatic treatment increases the contents of reducing sugars, soluble solids, and galacturonic acid and the titratable acidity of the final product. While medium- and high-temperature pectinases are widely used in industrial production, research and applications focusing on low-temperature pectinases remain limited. Compared with their medium- and high-temperature counterparts, low-temperature pectinases offer advantages such as reduced energy and cost requirements, minimal juice turbidity and bitterness, and preservation of nutrients and volatile flavor compounds in fruits and vegetables. These benefits contribute to the retention of the product’s natural qualities [30,31,32].

In food processing, many raw materials and finished products, such as fruit juices, jams, and beverages, are highly sensitive to temperature. Data suggest that most cold-adapted pectinases are polygalacturonases, which are typically optimized for acidic pH values and are mainly used in juice production [33,34]. However, there are no commercially available pectin lyases produced on an industrial scale. This study involved the extraction of metagenomes from deep-sea sediment samples, followed by identification and cloning of a low-temperature pectin lyase, Pel1Ba, with a total activity of 5100 U/mL. When expressed in *E. coli*, Pel1Ba exhibited high specific activity, robust expression levels, and broad temperature adaptability (10–50 °C). It retained more than 20% of its residual activity in the pH range of 2–4, making it a promising candidate for low-temperature juice processing. The high expression level of Pel1Ba contributes to reduced production costs, facilitating its application in juice production. The expression levels could be further improved through expression in *Pichia pastoris*.

At 20 °C, increasing the amount of Pel1Ba added to orange juice enhanced juice yield and clarity. Regarding clarity, 10 U/mL resulted in significant improvements compared to 0 U/mL and 5 U/mL, with no significant differences observed between 10 U/mL and 20 U/mL. Regarding juice volume, 10 U/mL showed significant improvements compared to 0 U/mL but not compared to 5 U/mL or 20 U/mL. Thus, 10 U/mL is the optimal enzyme dosage for a 10 mL juice system. Differences in performance may result from factors such as enzyme type, orange variety, and processing conditions. Determining the optimal enzyme ratio could further enhance juice clarification.

Pectinases have broad applications in juice extraction and clarification, where they effectively reduce juice viscosity and enhance transparency. Studies have demonstrated that treating fruit pulps (e.g., banana, grape, and apple) with pectinases significantly improves juice yield [35]. Furthermore, pectinases are employed to soften the albedo in citrus fruits, improving the production efficiency of canned oranges and replacing traditional manual processes [36]. Additionally, the combined use of different types of pectinases can improve juice clarification efficiency, and combining pectinases with amylases reduced the filtration time by approximately 50% [37]. In some cases, co-application with cellulases, arabinases, or xylanases further enhanced pressing efficiency [38]. Wang et al. [39] reported that when fruit juice was treated with both endo-polygalacturonase PGA-ZJ5A and pectin lyase PNL-ZJ5A, the transmittance increased by 18.1% compared to treatment with PGA-ZJ5A alone. This indicated that PNL-ZJ5A has the ability to react with oligogalacturonic acid, the enzymatic product of PGA-ZJ5A, which contributes to the improved transmittance of the juice. The addition of PNL-ZJ5A also had a similar impact on juice volume. When pear juice was treated with PGA-ZJ5A and PNL-ZJ5A, its volume increased from 6.06 mL (achieved with PGA-ZJ5A alone) to 6.53 mL within 2 min, representing a 7.7% increase. This demonstrates that the synergistic action of these two enzymes generated soluble substances during pear juice decomposition, thereby enhancing filtration efficiency. Compared to PGA-ZJ5A and PNL-ZJ5A, Pel1Ba improved the clarification rate of orange juice by 37.80%, surpassing their clarification effects. Therefore it is hypothesized that the pectate lyase Pel1Ba in the present study in combination with different types of pectinases may have better application in juice clarification process, which needs to be further investigated.

## 5. Conclusions

In summary, the low-temperature stable Pel1Ba from *Bacillus subtilis* maintains more than 80% of its maximum enzyme activity (5100 U/mL) under low-temperature conditions ranging from 0 to 40 °C. After treatment of freshly squeezed orange juice at 20 °C with Pel1Ba, the juice clarification effect was significantly enhanced, with a notable increase in juice volume. This demonstrates that Pel1Ba retains high enzyme activity under low-temperature conditions and shows specific potential for juice clarification. It is a pectate lyase with a broad temperature range, suitable for NFC (Not From Concentrate) fresh fruit cold-pressing processes and has promising applications in fresh juice clarification. Furthermore, through the Preoptem temperature model, a low-temperature model prediction was conducted for Pel1Ba with single-site mutations. The mutants A230I, F253I, and L292I showed significant increases in stability at 20 °C, with residual enzyme activity increasing by 22.5%, 34.4%, and 25.1%, respectively. These findings provide valuable insights for the low-temperature modification of pectate lyases.

## Figures and Tables

**Table 1:** Effects of metal ions and chemical reagents on Pel1Ba activity.

Metal Ion	Relative Activity (%)	
	1 mM	10 mM
control	100	100
Mg^2+^	30.20 ± 2.91	31.87 ± 0.98
Mn^2+^	132.27 ± 1.12	125.34 ± 1.52
Co^2+^	42.12 ± 1.17	40.58 ± 1.44
Cu^2+^	48.40 ± 1.99	54.46 ± 1.05
Fe^3+^	85.62 ± 2.64	2.80 ± 0.13
Fe^2+^	82.97 ± 2.73	57.48 ± 2.92
Ni^2+^	86.85 ± 0.33	87.38 ± 3.74
Zn^2+^	92.09 ± 0.74	93.63 ± 1.53
K^+^	123.49 ± 4.12	126.79 ± 1.67
Ca^2+^	130.36 ± 3.07	146.64 ± 3.67
EDTA	86.67 ± 0.32	40.62 ± 1.30
SDS	73.58 ± 2.36	33.45 ± 1.24

**Table 2:** Kinetic constants of the WT and the mutant enzymes.

Enzyme	Vmax (μmol·min^−1^·mL^−1^)	Kcat (s^−1^)	Km (mol/mL)	Kcat/Km (×10^2^ mL·mg^−1^·s^−1^)
Pel1Ba	0.02997	12.70	8.98	1.41
A230I	0.02861	13.95	8.56	1.63
F253I	0.01853	11.32	5.69	1.99
L292I	0.02641	15.08	8.11	1.86

## Data Availability

All data underlying the results are included as part of the published article and its Supplementary Materials.

## Supplementary Materials

The following supporting information can be downloaded at: https://www.mdpi.com/article/10.3390/microorganisms13030634/s1, Figure S1: Kinetic parameters of Pel1Ba toward citrus peel pectin; Table S1: ΔΔG (kcal/mol) predictions for single-point mutations of Pel1Ba; Table S2: Mutation sites and primers used for PCR.
