# Supplementary material for: A Low-Temperature-Active Pectate Lyase from a Marine Bacterium for Orange Juice Clarification
Source: Microorganisms. 2025 Mar 11;13(3):634. doi: 10.3390/microorganisms13030634 (PMC11944935; doi:10.3390/microorganisms13030634)
Supplement: Supplementary file 1 [file microorganisms-13-00634-s001.zip › microorganisms-3461292-supplementary.pdf]

supplementary material

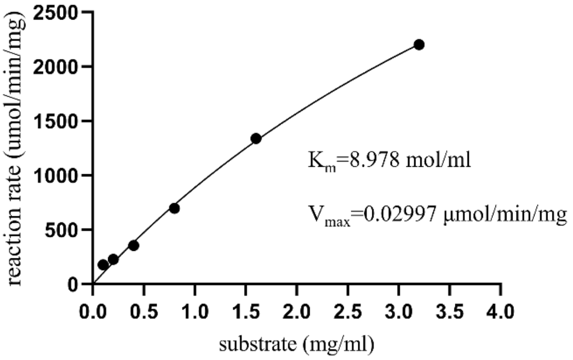

Figure S1. Kinetic parameters of Pel1Ba towards citrus peel pectin.

Table S1. ΔΔG (kcal/mol) predictions for single-point mutations of Pel1Ba.

Mutation	ΔΔG (kcal/mol)
N215A	-0.695745
T301K	-0.696191
G302K	-0.696344
S213N	-0.697331
L292V	-0.698837
A230V	-0.699095
L87F	-0.699715
F253I	-0.700687
L292I	-0.700819
A230I	-0.701544
N215K	-0.701775
A37V	-0.702798
T337K	-0.710678
K89A	-0.720797

Table S2. Mutation sites and primers used for PCR.

Primer	sequence (5`-3`）	Tm (℃)
F253I-F	CTAGCATCaTCGGCTCTTCCGATAGCAAAACT	64.5
F253I -R	AGAGCCGAtGATGCTAGATTTATCGTGATCGTGA	60.6
L292I-F	GGCCAGGTTCATatcTATAACAACTATTACGAGGGTTCTACTGG	61.1
L292I-R	TAgatATGAACCTGGCCGAAACGCACGCGTGG	81.5
A230I-F	GACCGACatTAGCAATGGTGCTAACTATATCACCA	60.6
A230I-R	CCATTGCTAatGTCGGTCTGACCATCATGATGC	62.3
N215K-F	TCTCCGAAgTACTTCGGCCGCAAATACCAGCA	68.6

N215K-R	CCGAAGTAcTTCGGAGAAGTGGAATCCGGACG	69.2
A37V-F	TTACACCGtTTCCAACCGCAACCAGCTGGTTA	68.5
A37V-R	GGTTGGAAaCGGTGTAAACCTGAGAAGAAGATG	60.1
T337K-F	GGCAGCAAAAAagGTGTCTGTGTTTTCCGGTGGTAC	61.0
T337K-R	GACACctTTTTTGCTGCCAGCAGACCTGGAACG	74.0
K89A-F	GACCTGAACgcATATCTGAAAGCATACGACCCGA	60.8
K89A-R	CAGATATgcGTTCAGGTCGTATTCCGGATCCTT	64.8
